# A YoeB toxin cleaves both RNA and DNA

**DOI:** 10.1038/s41598-021-82950-6

**Published:** 2021-02-11

**Authors:** Julia McGillick, Jessica R. Ames, Tamiko Murphy, Christina R. Bourne

**Affiliations:** 1 Department of Chemistry and Biochemistry, University of Oklahoma, Norman, OK 73019 USA; 2 Present Address: GENEiQ, Dallas, TX USA; 3 Present Address: School of Physics, University of Bristol, Bristol, England; 4 Present Address: Baylor College of Medicine, Houston, TX USA

**Keywords:** Nucleases, Applied microbiology

## Abstract

Type II toxin-antitoxin systems contain a toxin protein, which mediates diverse interactions within the bacterial cell when it is not bound by its cognate antitoxin protein. These toxins provide a rich source of evolutionarily-conserved tertiary folds that mediate diverse catalytic reactions. These properties make toxins of interest in biotechnology applications, and studies of the catalytic mechanisms continue to provide surprises. In the current work, our studies on a YoeB family toxin from *Agrobacterium tumefaciens* have revealed a conserved ribosome-independent non-specific nuclease activity. We have quantified the RNA and DNA cleavage activity, revealing they have essentially equivalent dose-dependence while differing in requirements for divalent cations and pH sensitivity. The DNA cleavage activity is as a nickase for any topology of double-stranded DNA, as well as cleaving single-stranded DNA. AtYoeB is able to bind to double-stranded DNA with mid-micromolar affinity. Comparison of the ribosome-dependent and -independent reactions demonstrates an approximate tenfold efficiency imparted by the ribosome. This demonstrates YoeB toxins can act as non-specific nucleases, cleaving both RNA and DNA, in the absence of being bound within the ribosome.

## Introduction

Toxin-antitoxin systems are widespread in prokaryotes, with many studies focused on the Type II systems comprised of a protein toxin and a tightly interacting but labile protein antitoxin^1–4^. TA systems function analogously to kin-recognition and contact-dependent inhibition systems, wherein the toxin activity is deleterious to cells while the antitoxin serves an “immunity” function^5^. TA systems differ from these other toxin-immunity systems, however, in remaining intracellular and thus the toxin functions directly on the native host cell^6,7^. While their role in bacterial physiology is uncertain^8–11^, TA systems can be co-opted as useful tools in biotechnology. Examples include selection markers on plasmids and in applications of expression in eukaryotic cells^12–17^.

Toxins in the Rel-superfamily are grouped based on structural homology and include sub-families denoted as HigB, YafQ, YoeB, and RelE; toxins in each of these sub-families cleave mRNA within the ribosomal A-site, although the specific catalytic mechanisms are divergent^18–23^. Briefly, the toxin will bind to a ribosomal subunit, interacting with the A site and either preventing formation of the translation initiation complex or carrying out sequence-specific mRNA cleavage^21–25^. We have recently identified a YoeB toxin from *Agrobacterium tumefaciens*, herein referred to as AtYoeB, that also is a ribosomal-independent mRNase consistent with some previously characterized YoeB toxins^21,26,27^. During these studies with AtYoeB we noted an additional catalytic activity, the cleavage of DNA, which is not previously characterized for this protein family or fold.

In the current work we report on the pervasive nuclease functions of AtYoeB and directly compare RNA and DNA cleavage in solution. When compared to ribosome-dependent RNA cleavage, the ribosome-independent nuclease activity using either RNA or DNA as substrates are essentially equivalent and tenfold less efficient than when the ribosome is present. Additionally, we identify that the DNA degrading activity is dependent on divalent cations, pH-dependent, and is functional on one strand of a double-stranded DNA independent of topology, as well as on single-stranded DNA. The dimeric AtYoeB toxin interacts with double-strand DNA yielding two independent binding events with affinities (K_D_) in the micromolar range. These results highlight that this fold is able to function as a non-specific nuclease, with the ribosome improving the efficiency of catalysis while also restricting the substrate to mRNA.

## Results

### AtYoeB cleaves both DNA and RNA in vitro with equal efficiency

**Figure 1:**
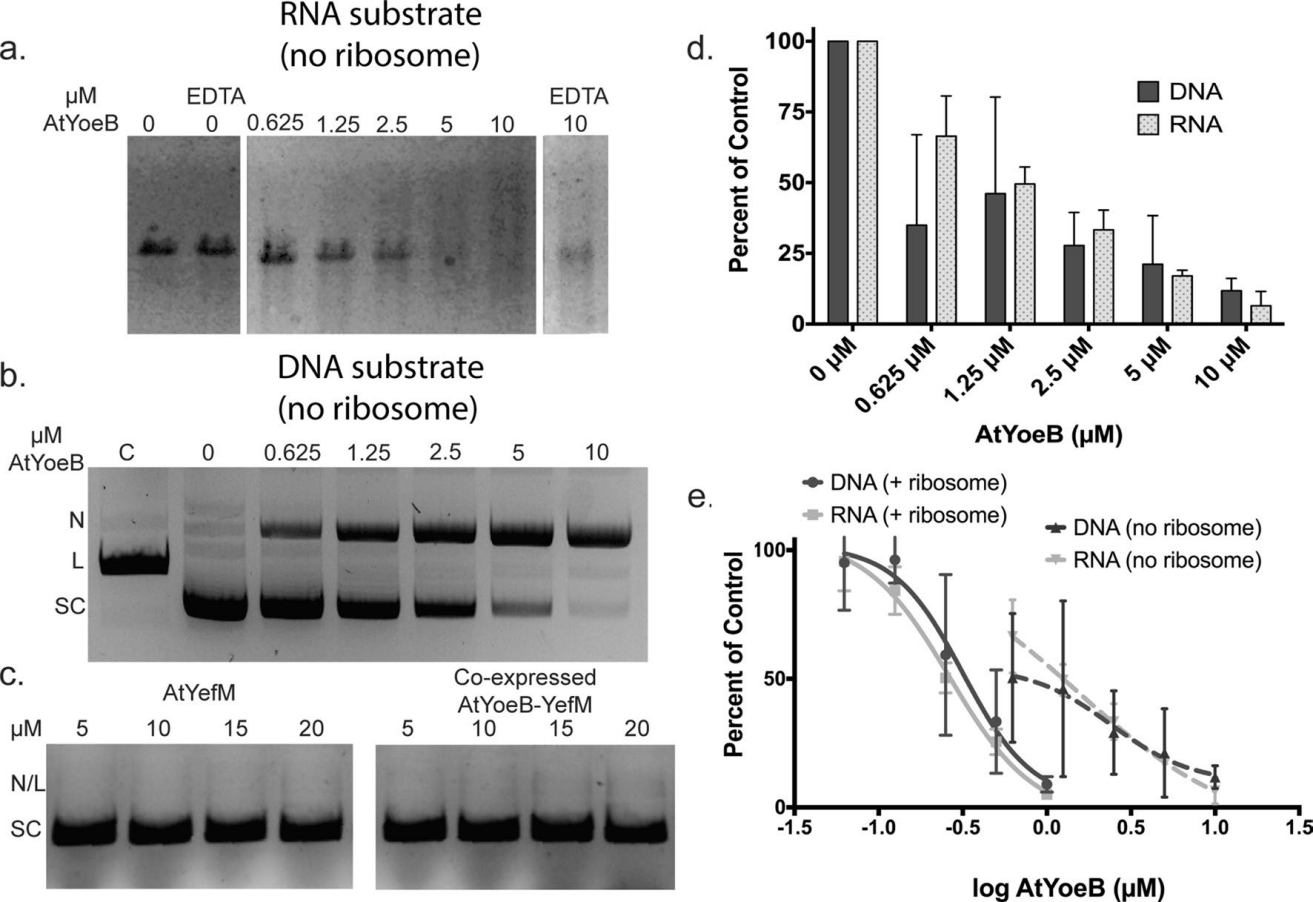
The AtYoeB toxin cleaves both RNA and DNA in vitro with the same concentration dependent efficiency. (**a**) AtYoeB possesses a sequence-independent RNase activity when incubated at 37 °C for 30 min; cleavage progresses in the presence of 2.5 mM MgCl_2_ (replicates given in Fig. S1), and while EDTA appears to slow the reaction, AtYoeB still degrades approximately 65% of the starting substrate indicating a metal-independent activity. (**b**) AtYoeB degrades DNA in a dose-dependent and sequence-independent manner, resulting in the accumulation of a nicked (N) topology under these reactions conditions. (SC, supercoiled; L, linear) (**c**) No DNA cleavage activity is present for the antitoxin AtYefM or for the complex of AtYoeB-YefM, demonstrating that DNA cleavage activity resides with the AtYoeB toxin. (**d**) Reactions performed using either DNA or RNA substrates in the presence of 2.5 mM MgCl_2_ and increasing AtYoeB were analyzed by electrophoretic methods (as in panels (**a**) and (**b**)). The intensity of either supercoiled DNA (*n* = 3) or total RNA (*n* = 3) were measured after electrophoresis and normalized relative to the control sample with no AtYoeB. (**e**) Quantitation of the dose dependence of RNA and DNA degradation in the presence of ribosomes (in vitro coupled transcription-translation) and absense of ribosomes reveals a much less efficient cleavage without the ribosome.

We have confirmed that the AtYoeB toxin is also able to cleave RNA in vitro in the absence of the ribosome (Fig. 1a, additional gels in Fig. S1). During these studies, however, we noted that AtYoeB is also able to mediate DNA degradation in vitro (Fig. 1b), while the corresponding antitoxin AtYefM or complexed AtYoeB-YefM do not cleave DNA (Fig. 1c). Analysis of the reactions with either RNA or DNA as substrates demonstrates that the EC_50_, defined as the effective concentration of AtYoeB at which 50% of the initial substrate is lost, is similar regardless of the type of nucleic acid template (Fig. 1d). In the absence of ribosomes, the EC_50_ for DNA substrate is 2.4 ± 0.37 µM, while for an RNA substrate it is 1.5 ± 0.17 µM. These cleavage reactions are much more efficient when the ribosome is present, presumably due to a previously noted ordering of the YoeB active site C-terminal residues as well as the stablization of the mRNA substrate^21^. Using an in vitro coupled transcription-translation system we determined the EC_50_ value for AtYoeB to range from 0.26 to 0.32 µM^27^ (Fig. 1e), with essentially no differences in activity using either RNA or DNA as the initiating species in this coupled reaction kit.

### AtYoeB DNA cleavage activity is pH- and cation-dependent, and can act on any topology of DNA

**Figure 2:**
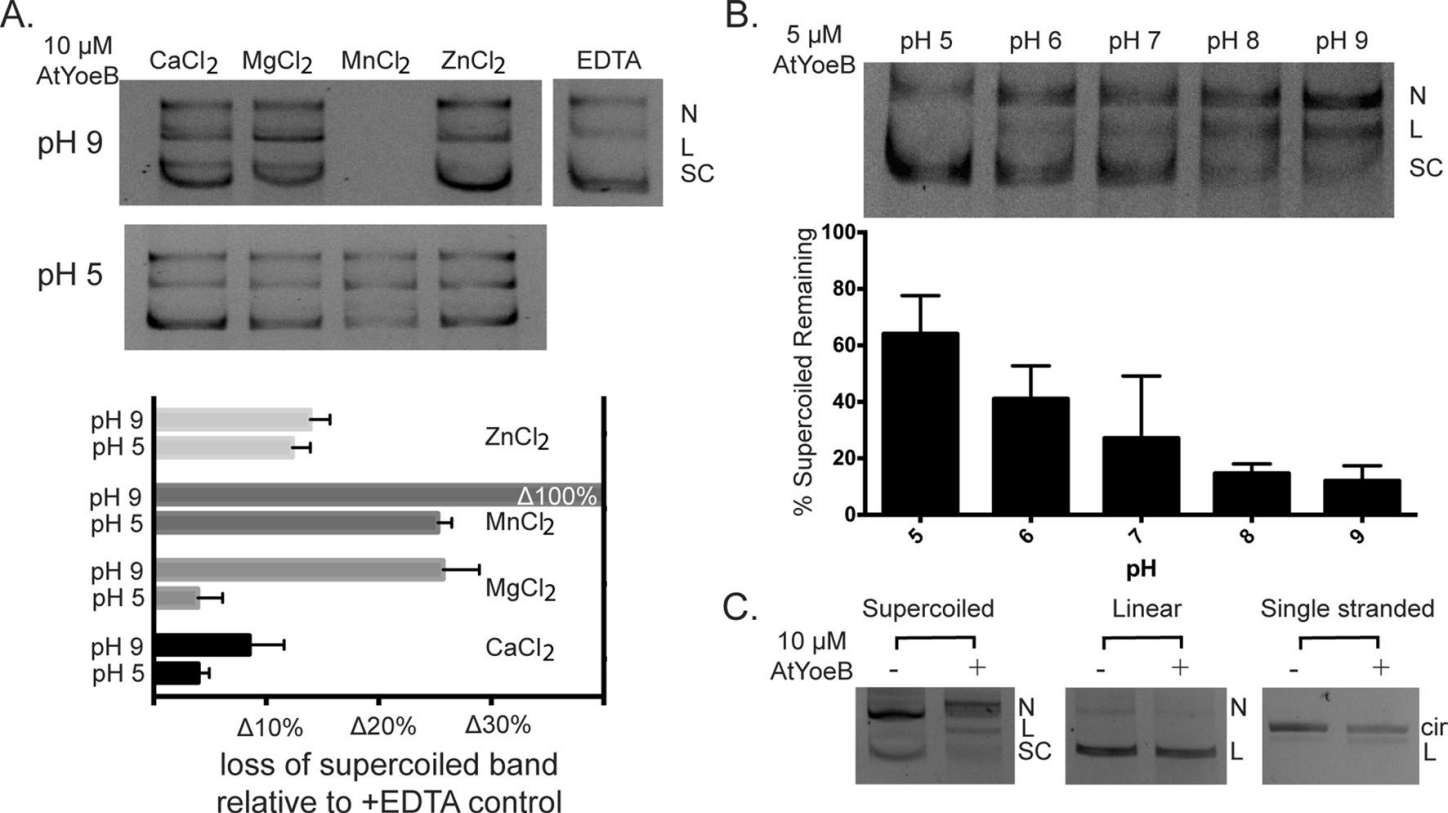
The in vitro DNA cleavage activity of AtYoeB requires a divalent cation, is pH sensitive, and has no preference for substrate topology. (**A**) DNA cleavage requires a divalent cation, with manganese producing the most efficient reaction such that at pH 9 the DNA substrate is completely degraded, while at pH 5 approx. 30% is degraded (n = 6, also see Fig. S2). Magnesium is also sufficient, again with increased degradation at pH 9 versus pH 5. Zinc and calcium do not show strong pH dependence and are not as efficient at mediating catalysis. (supercoiled (SC), nicked (N) and linear (L) topologies denoted) (**B**) DNA cleavage assays were carried out at different pH values, and the resulting intensity of the remaining supercoiled toplogy was quantified (n = 3, also see Fig. S3). A marked pH dependence is noted, with activity present at pH 5 and 6, but with the highest activity measured at pH 9. (**C**) The type or topology of the starting DNA substrate did not affect the resulting cleavage, with approx. 25–35% decrease in total intensity for each type of DNA substrate after incubation with AtYoeB (n = 3, also see Fig. S5).

The ability of AtYoeB to cleave DNA was determined to be metal-dependent, with both magnesium and manganese able to robustly support the catalytic activity (Figs. 2A and S2 for additional gel images and calculations). Calcium and zinc, however, seemed to not be used by the toxin to mediate effective catalysis. This metal dependence was further confirmed by an absence of DNA cleavage in the presence of EDTA (Fig. S2).

As seen in Fig. 1a, RNase activity is not a metal-dependent reaction, but does proceed faster in the presence of magnesium. This is consistent with proposed mechanisms that rely on the nucleophilic activity of the 2′-OH of RNA in concert with the general acid and base residues of the protein. Previous studies identified the catalytic residues for YoeB toxin degradation of RNA as Glu46, acting as a general base, and the C-terminal His residue (amino acid 83 in Ec, and amino acid 87 in At) acting as a general acid^21,28^. This mechanism indicates that the histidine must be protonated, imparting sensitivity to pH for the reaction. We predicted that these amino acids, including the histidine, would also be utilized for the degradation of DNA. Consistent with this expectation, there was a sensitivity of DNA degradation to pH. However, the maximum DNA degradation was noted around pH 8–9, where a histidine would be fully deprotonated, as expected to promote interaction with cation(s) (Figs. 2B and S3, which includes additional gel images and calculations). At pH 9, potent DNA degrading activity is noted, resulting in only 12% of the starting DNA remaining supercoiled. At pH 7, an average of around 30% of the DNA remained supercoiled, while prominent bands for both nicked and linear DNA substrate are visible and increase as the pH increases. Further studies note no impact of these pH values on the overall stability of the AtYoeB toxin protein (Fig. S4). To assess the impact on catalysis, the pH was varied as was the cation used. The metal ion manganese catalyzed a complete loss of DNA substrate at pH 9; however, at pH 5 this was slowed to result in a loss of approx. 25% of the supercoiled band (Fig. 2A). A similar relationship was observed with magnesium, although the extent of degradation was tempered relative to manganese, with a loss of approx. 27% at pH 9 and only approx. a 5% loss at pH 5.

AtYoeB DNA cleavage activity can also be detected on linear and single-stranded substrates (Fig. 2C). The total intensity of DNA measured in each lane was used to assess linear and single-stranded circular substrate degradation. The samples with AtYoeB were then compared to the same samples lacking the toxin to yield a percentage of remaining DNA substrate. Little variation was noted between substrates (see Fig. S5 for additional gel images and calculations) with an average remaining DNA substrate across all those tested of 70 ± 6.8% under these reaction conditions. We note that, for the linear plasmid substrate no accumulation of smaller products was noted that would indicate a preferred site or sequence for the cleavage. This supports a lack of specific sequence recognition that would yield distinct products, and instead the loss of linear substrate is distributed among many sizes of smaller products that are not of sufficient concentration to be detected in this assay.

### AtYoeB-mediated DNA cleavage is blocked by its intearction with cognate antitoxin AtYefM

**Figure 3:**
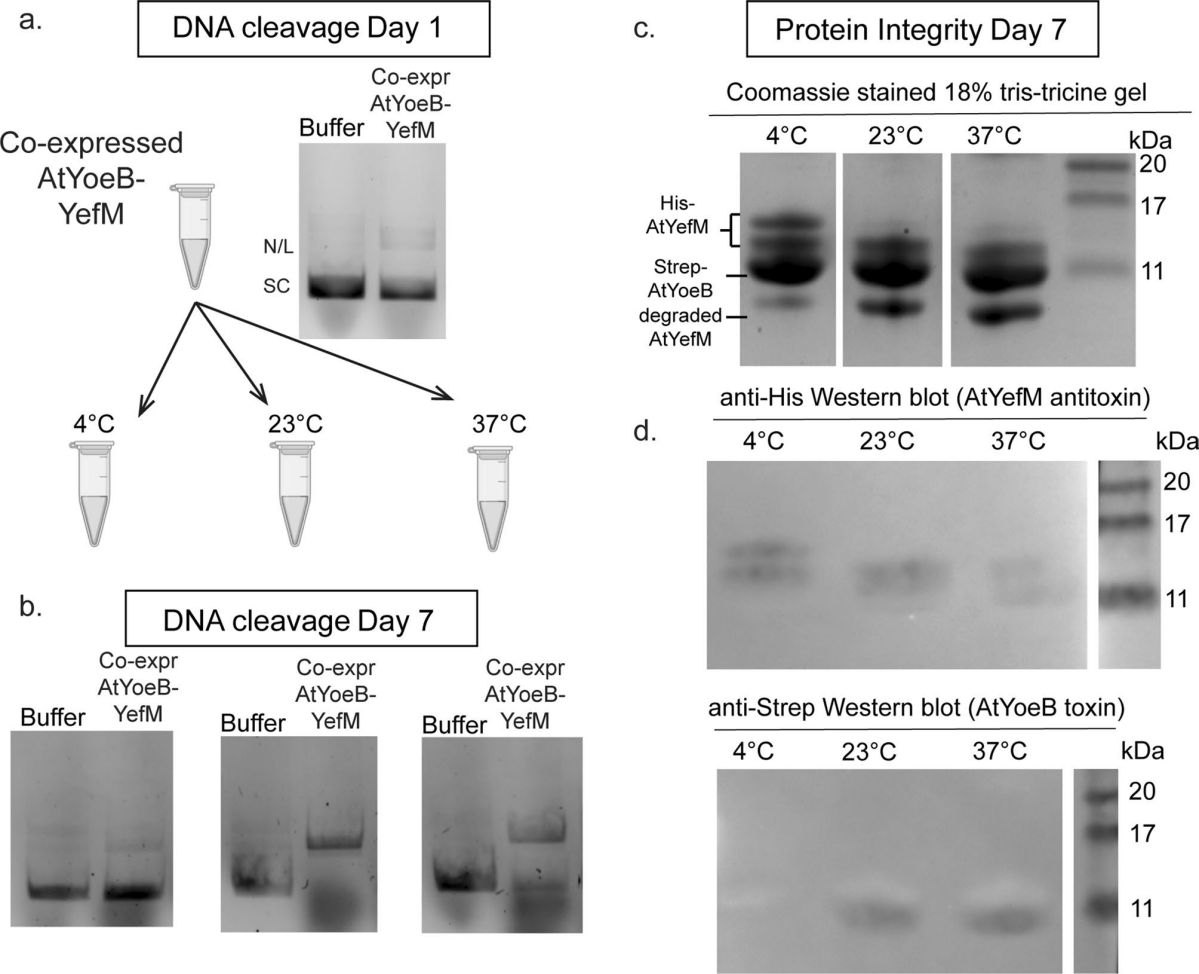
The AtYefM antitoxin blocks AtYoeB-mediated DNA cleavage, but it readily undergoes degradation that releases AtYoeB catalytic activity. (**a**) DNA degradation is not present for 10 µM co-purified AtYoeB-YefM complex immediately after purification. (**b**) After incubation for 1 week at temperatures above 4 °C, however, DNA cleavage activity is apparent. (**c**) Electrophoretic analysis of the protein samples used in panel (**b**) reveals degradation of one of the protein components that increases after storage at 23 °C and 37 °C (relative to 4 °C). Resolution of the doublet for intact AtYefM antitoxin requires a high percentage (18%) tris-tricine acrylamide gel. (**d**) Western blots were used to identify the individual bands visualized in the gel in panel (**c**), revealing that the degraded component is the His-tagged AtYefM antitoxin, while the Strep-tagged AtYoeB toxin remains unchanged. Note that the lowest band that accumulates is likely degraded AtYefM antitoxin, which appears to no longer carry the N-terminal His tag. (Full images of blots are provided in Fig. S6).

Experiments were carried out to verify that the observed DNA cleavage arises directly from catalytic activity of the AtYoeB toxin. In initial experiments, we observed dose-dependent degradation of DNA only for purified AtYoeB; no DNA degradation was observed for reactions containing purified AtYefM or the co-purified AtYoeB-YefM complex (Fig. 1b,c). However, some DNA cleavage was noted for the AtYeoB-YefM complex but only after prolonged (> 2 weeks, 4 °C) storage. This indicated that the labile antitoxin was potentially degraded from the complex, thus freeing the toxin’s catalytic activity. To test this idea, purified co-expressed AtYoeB-YefM were prepared and immediate analysis again demonstrated no significant DNA degrading activity (Fig. 3a). From this solution, we incubated identical aliquots of the purified protein complex at 4 °C, 23 °C, and 37 °C. After one week, these samples were again analyzed for DNA cleavage activity (Fig. 3b). The protein integrity was also assessed by visualization on Coomassie-stained gels (Fig. 3c), as well as using Western blots to detect the Strep-tagged toxin and His-tagged antitoxin (Fig. 3d). After this one week incubation, DNA cleavage activity was prominent in the 23 °C and 37 °C samples, and Western blotting confirmed antitoxin degradation. In this co-expressed complex, the antitoxin retains an N-termial His affinity tag; multiple protein bands reacting with the anti-His antibody are evident after storage at 23 °C and 37 °C, as well as the appearance of an additional low molecular weight band on the Commassie-stained gels that lacks reactivity on Western blots which we interpret as degraded antitoxin protein that has lost this affinity tag. In contrast, the sample stored at 4 °C had much less DNA cleavage acitivity, and this correlated with less antitoxin degradation visible in the electrophoretic analysis. These experiments verified that the Strep-tagged toxin remained intact, as the resulting bands indicate no changes in mobility or reactivity. These results clearly demonstrate a gain of DNA cleavage activity mediated by AtYoeB concomitant with degradation of the AtYefM antitoxin.

### The AtYoeB toxin can bind to DNA

**Figure 4:**
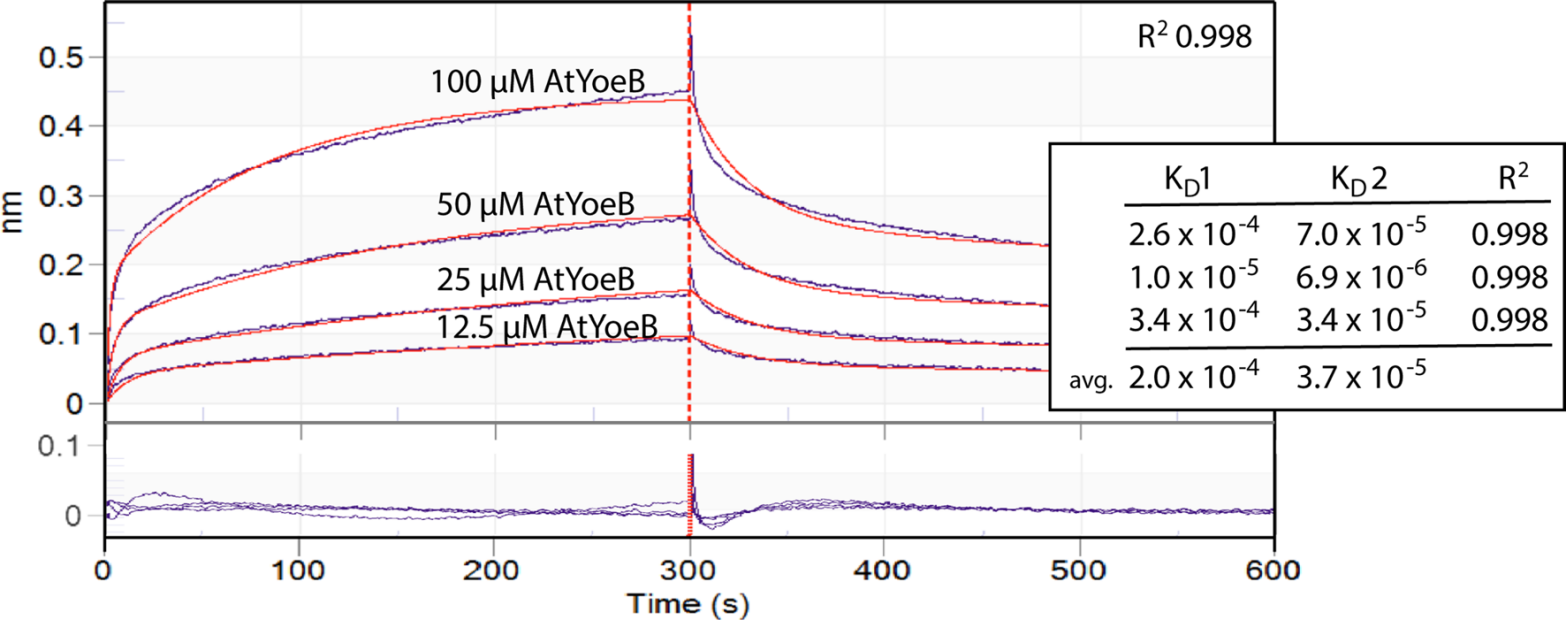
Biolayer interferometry was used to monitor the interaction of AtYoeB with immobilized double-stranded DNA, revealing two independent binding events (*n* = 3, 2:1 stoichiometric fit). A constant amount of biotinylated double-stranded DNA (232 bp) was immobilized on streptavidin-modified BLI pins (ForteBio) that were then incubated with different concentrations of AtYoeB. The resulting association and dissociation rates were used to fit the data to a 2:1 binding model (red lines), which is consistent with dimeric YoeB toxin interacting with DNA. The residual of the fit (purple lines) is shown below the binding curves.

To further validate the DNA cleavage by AtYoeB, we sought to demonstrate if it was able to bind to DNA and further, to measure how stongly they interact. Utilizing Biolayer Interferometry (BLI), the direct association and disassociation of AtYoeB toxin was visualized as it interacted with a 232 bp fragement of DNA (Fig. 4). The resulting kinetic rates were not adequately fit by a 1:1 interaction model, instead requiring a 2:1 fit to the data. This complex binding stoichiometry is unlikely to have arisen from non-specific interactions, as the concentration of protein is low compared to the resulting K_D_ value(s), the loading of the DNA is not close to saturation of the individual tips, and no interactions are present for AtYoeB binding to biocytin-blocked pins. It is likely that these two linked binding events arise due to the double-stranded DNA substrate interacting with dimerized AtYoeB toxin. The resulting measurements of K_D_ reveal an approximate tenfold difference between the two sites, with the weaker binding producing 200 µM affinity and the stronger site closer to 37 µM. Given the apparent lack of recognition of specific nucleotide base sequences, these affinity magnitudes appear reasonable and the difference between the binding events likely results from steric hinderance. Overall, these data demonstrate the feasibility for the YoeB toxin fold to interact with DNA, an obviously necessary step in the subsequent enzymatic cleavage.

## Discussion

During characterization of canonical YoeB functions, we have verified AtYoeB can carry out RNA cleavage in the absence of ribosomes. Precedence exists for this ribosome-independent RNase activity, which has also been observed in vitro for both *Escherichia coli* and *Staphylococcal aureus* YoeB^21,26^. Quantitation of the *S. aureus* YoeB RNase reaction yielded a half-maximal concentration of 2.4 µM YoeB, which is quite close to the 1.5 µM for RNA (and 2.4 µM for DNA) for AtYoeB measured in the current work. This residual ribosome-independent RNase acitvity is likely a property of the overall tertiary fold of the YoeB toxins, wherein catalytic resides at the most C-terminal portion of the protein that would be mobile in solution^21^. In the ribosome-free state these amino acids are able to sample multiple conformations, including fluctuations through a catalytically competent one; in contrast, this catalyically competent conformation is locked into place by docking within the ribosomal A site. Others have also noted structural similarities between the RelE superfamily, which includes YoeB, and that of well characterized ribosome-independent RNases including the BrnT and MqsR toxins^29,30^, and Barnase and Rnase Sa^26^. Therefore, it is not surprising that we find that the YoeB toxin from *A. tumefaciens* is able to cleave RNA in both ribosome-dependent^27^ and -independent reactions. The current study provides a unique contribution by directly comparing RNA cleavage with and without ribosomes, such that we can quantify the role of ribosome in AtYoeB functions. This highlights an almost tenfold increase in efficiency of translational inhibition versus cleavage of RNA in vitro in the absence of the ribosome. Further, because the dose-dependence of translation inhibition is the same in this coupled transcription-translation system regardless of starting with a DNA or RNA substrate, we can conclude that the ribosomal-dependent translational inhibition must be the dominant reaction.

In the current study we also discovered that AtYoeB is also able to cleave DNA with an efficiency comparable to the ribosome-independent RNase activity, and that the DNA cleavage reaction is both metal- and pH-dependent. The observed DNA cleavage reaction appears limited to a nickase function when the substrate is double-stranded, as a supercoiled substrate is quickly converted to nicked forms. At longer times and higher concentrations of AtYoeB, a linear DNA topology accumulates and then disappears altogether at longer timepoints as it is fully fragmented in a non-specific manner. The nickase function is also supported by the ability of AtYoeB to cleave ssDNA and linear forms with equal efficiency.

While DNA cleavage by AtYoeB is detectable at concentrations as low as 156 nM (Fig. S7), within the cell it is likely that the available antitoxin-free YoeB would be quickly complexed with ribosomes that then impart a requisite specificity for mRNA. Previous accounts demonstrated a relatively high affinity for RNA within a ribosomal context and the presence of the ribosome appears to order the C-terminal catalytic residue^23,31^, which we have now determined yields an activity 10 times that observed in ribosome-independent reactions^27^. This observation can be further coupled to an estimate of one YoeB molecule per five ribosomes under normal cellular conditions^31^. This diminishes the likelihood of AtYoeB encountering DNA within the cellular context, and thereby explains why cells expressing YoeB toxins do not exhibit characteristic morphological changes associated with the accumulation of DNA breaks. It should be noted, though, that biotechnology applications utilizing expression of *Streptococcus pneumonia* YoeB caused cell death of *Arabidopsis thaliana* via an unkonwn mechanism but with hallmarks of DNA damage^14^, further supporting our observations for in vitro DNA cleavage by AtYoeB.

As part of this study, it was important to demonstrate that this was arising directly from the toxin. The co-expressed complex of AtYoeB and AtYefM form a productive complex that is devoid of DNA cleavage activity. We are able to utilize time and temperature to promote loss of antitoxin from the complex, and in so doing the samples of residual AtYoeB clearly gain DNA cleavage activity. The gain of DNA cleavage activity correlates with the visible degradation of antitoxin, demonstrating that the AtYoeB toxin is capable of direct DNA degradation in vitro.

These findings indicate that the AtYoeB toxin, and likely other YoeB toxins, can function as “non-specific” nucleases in vitro*.* There are many examples of non-specific nucleases, which by definition can cleave RNA or DNA (single- or double-stranded)^32^. An approx. 10 kDa protein from *Helicobacter pylori*, HP0268, is a nuclease documented to exhibit both DNA nicking and RNase activity, very similar to our observations, although it is structurally similar to MutS and no structural homology is noted between this protein at AtYoeB^33^. Other studies have identified a DnaD-like domain in thermophilic phage with both DNase and RNase activity^34^. Further, a toxin structurally related to YoeB has been published as possessing DNA nickase activity, although no RNase activity was noted^35^. It is striking that both RNA nuclease and DNA nicking activity has been identified for the VapD toxin family^33^. The active site residues are shared by both nuclease activities, with acidic residues serving as the catalytic residues that coordinate a metal binding site. This mechanism was noted to be pH dependent, with increased activity above pH 8, similar to what is described in the current study. However, analysis of the structures of VapD and YoeB reveal no structural homology. These metal-independent RNases rely on the 2′-OH for the initial nucleophilic attack, which we surmise is replaced by interactions with a divalent cation to carry out the DNA cleavage function observed in AtYoeB. In conserved metal-dependent DNase folds, the metal is typically coordinated by a His amino acid that imparts a pH-sensativity^32^ as we have also observed for AtYoeB. Other studies have noted the conservation of the RelE/ParE fold, including homology with other RNase proteins including colicins and barnase^21,29,36^. The recognition of this deoxyribonuclease activity as a similar and potentially overlapping mechanism with the RNase ability highlights the plasticity of the conserved fold found in YoeB toxins.

## Materials and methods

### Cloning, expression, and purification for in vitro assays

The genes for the toxin (AtYoeB) and antitoxin (AtYefM) were amplified from *Agrobacterium tumefaciens* genomic DNA by PCR and placed into over-expression vectors using restriction mediated cloning techniques, as previously published^27^. In brief, the AtYoeB toxin was placed in both a pET-28a vector upstream from an inserted HRV3c protease site, GST fusion tag, and 6 × His affinity tag, as well as in the second multiple cloning site of a pET-Duet vector downstream of a constructed Strep-II affnity tag. The YefM antitoxin was cloned into the pET-15b vector in-frame with the 6 × His affinity tag and thrombin cleavage sequence, as well as within the first multiple cloning site of the same pET-Duet vector and also in frame with an N-terminal 6 × His affinity tag. These constructs allowed preparation of either the AtYoeB toxin or AtYefM antitoxin individually, or the complex of these proteins formed during co-expression from the pET-Duet vector.

Protein expression and purification was carried out as previously described^27^. In brief, BL21 DE3 *E. coli* were induced at 20 °C using 1 mM IPTG, and incubated overnight for AtYoeB (which is not overtly toxic to *E. coli*^27^) or the complex, and less than four hours for AtYefM antitoxin in the absence of AtYoeB to minimize degradation. Recombinant proteins were soluble under these expression conditions and were purified using nickel resin (Ni-NTA) for Immoblized Metal Affinity Chromatography. When necessary (such as for BLI measurements), affinity tags were removed with the appropriate proteases by incubation overnight at 4 °C, followed by a second purification over Ni-NTA resin to ensure removal of uncleaved protein. The final step of each purification was a size exclusion through a Sephadex-S75 column (GE Healthcare) equilibrated in Hepes pH 7.5, 150 mM NaCl. Purity was verified by tris-tricine electrophoresis (12% for analysis of purity, and 18% acrylamide for analysis of degradation) as visualized by Coomassie staining, and protein concentrations were calculated using extinction coefficients based on the amino acid content^37,38^.

### Western blots

Western blots were carried out using a semi-dry transfer method from tris-tricine gels to PVDF membranes with a 0.2 µm pore size. Blocking of non-specific sites utilized 1% non-fat milk in tris-buffered saline (TBS), and washing steps utilized TBS plus 0.1% Tween-20. Primary antibodies were an anti-pentaHis mouse antibody (1:5000 dilution in 1% milk-TBS, Qiagen) and an anti-Strep mouse antibody (1:3000 dilution in 1% milk-TBS, IBA Lifesciences), while the secondary antibody was a fluorescently labeled goat anti-mouse antibody (1:5000 dilution in TBS, BioRad Laboratories). Blots were imaged using a ChemiDoc system (BioRad Laboratories).

### RNA synthesis

RNA was synthesized using a HiScribe T7 High Yield RNA Synthesis kit (New England Biolabs) following manufacturer’s directions. The DNA template consisted of a T7 promoter upstream from the open reading frame for Firefly Luciferase (Fluc, control vector provided with the synthesis kit) or Green Fluorescent Protein (GFP, pET28a::GFP was a figt from Matthew Bennett, Addgene plasmid #60,733). The Fluc RNA was used for monitoring of RNase activity, while the GFP RNA was used as the template for in vitro translation assays.

Resulting RNA was purified by standard phenol-chloroform extraction, treated with DNase I (New England Biolabs) and recovered by ethanol precipitation. RNA was re-suspended in nuclease-free water, its absorbance at 260 nm was measured and used to calculate concentration, and the samples stored at − 20 °C until use.

### Ribosome-dependent assays

A commerical coupled transcription-translation kit was utilized (PURExpress In Vitro Protein Synthesis Kit, New England Biolabs) following manufacturer’s directions. Reactions were incubated with increasing concentrations of purified AtYoeB protein and either 300 ng linearized plasmid DNA or 7.5 µg of synthesized RNA. Monitoring of translation was achieved by measuring the fluorescent signal arising from Green Fluorescent Protein (ex 485 nm, em 528 nm) over the course of 2 h at 37 °C.

### Gel-based DNase assays

Reactions to measure the ability of a toxin to cleave DNA were assembled by combining equal volumes of 500 ng plasmid pBR322 (purified from *E. coli* using commercial miniprep kits), 2 mM MgCl_2_, and water with 10 µL of a 2 × concentration of purified AtYoeB, resulting in 20 µL assay volumes. Reactions were incubated at 37 °C for 30 min, mixed with SDS to a final concentration of 1%, and then incubated at 50 °C for 15 min. Samples were mixed with loading dye and electrophoresed on a 1% agarose TAE gel containing SYBR safe at 80 V for 30 min.

To test pH, substrate, or metal dependence, a final concentration of 5 or 10 µM toxin, as noted, was added to the reaction, and the relevant solutions were varied appropriately. In the case of the pH dependence assay, Bis-Tris buffer at pH 5 or 6 or Tris-HCl at pH 7, 8 or 9 was added to a final concentration of 100 mM. For analysis of topology preferences of DNase activity, substrates included supercoiled pBR322 (from a miniprep kit), linearized pBR322 (obtained from restriction digests and further purified), or the single-stranded M13 genome (New England Biolabs). In the case of the metal dependence assay, MgCl_2_, MnCl_2_, CaCl_2_, or ZnCl_2_ were added to a final concentration of 2 mM. Product bands were visualized with a ChemiDoc unit (Bio-Rad) and quantified with ImageJ^39^.

### Gel-based RNase assay

The ability of AtYoeB to cleave RNA in the absence of the ribosome was measured using a gel-based assay. Reactions contained a final concentration of 80 ng Fluc RNA, 0.625 μM to 10 μM AtYoeB, 150 mM NaCl, and 2.5 mM MgCl_2_ or 5 mM EDTA to chelate metal cations. Reactions were incubated at 37 °C for 30 min, after which, the reactions were mixed with formaldehyde loading dye (Lonza Bioscience). Samples were then loaded onto a 1.2% FlashGel™ RNA cassette (Lonza Bioscience) and electrophoresed at 275 V for 8 min using the Lonza FlashGel™ kit. Product bands were visualized with the manufacturer provided software and quantified with ImageJ^39^.

### Biolayer interferometry (BLI) assay

DNA was amplified using biotinylated forward and reverse primers to the “strong gyrase site” found in the pBR322 plasmid (positions 867 to 1070)^40,41^, and purified from the resulting reactions using a PCR clean-up spin column kit (Promega Corporation). All solutions were prepared to result in a final 1 × composition of 0.5%BSA, 0.05%Tween-20 in 50 mM Tris-HCL pH 8.5 and 150 mM NaCl. Biotinylated DNA (232 bp, 125 nM) was captured onto Super Streptavidin biosensors (FortéBio) using an Octet Red model instrument (FortéBio). Dilutions of AtYoeB toxin protein were incubated with captured DNA until saturation to obtain the on rate for association, then the complex was incubated in buffer to obtain the off rate for disassociation. Data were processed with the FortéBio Octet Data Analysis software using best practices, yielding valid fits to the observed data with a model of 2:1 stoichiometry.

## Supplementary Information

Supplementary Figures.
