# Supplementary material for: A YoeB toxin cleaves both RNA and DNA
Source: Sci Rep. 2021 Feb 11;11:3592. doi: 10.1038/s41598-021-82950-6 (PMC7878887; doi:10.1038/s41598-021-82950-6)
Supplement: Supplementary file 1 — Supplementary Figures. [file 41598_2021_82950_MOESM1_ESM.pdf]

Supplementary Material

A YoeB toxin cleaves both RNA and DNA

Julia McGillick[1,2], Jessica R. Ames[1,3], Tamiko Murphy[1,4], and Christina R. Bourne[1*]

[1]Department of Chemistry and Biochemistry, University of Oklahoma, Norman, OK, 73019, USA

*Corresponding author information. Tel: +01 (405) 325-5348; Fax: +01 (405) 325-6111; Email: cbourne@ou.edu

[2]Present Address. GENEiQ, Dallas Texas, USA

[3]Present Address. School of Physics, University of Bristol, Bristol, England

[4]Present Address. Baylor College of Medicine, Houston Texas, USA

Figure S1. Replicates for the dose dependence of the observed AtYoeB-mediated RNase activity in the absence of the ribosome

Figure S2. Replicates and calculations for the divalent cation dependence of the observed AtYoeB-mediated DNase activity.

Figure S3. Replicates and calculations for the pH dependence of the observed AtYoeB-mediated DNase activity demonstrate the highest activity is at pH 7 and greater.

Figure S4. AtYoeB protein is stable at least up to pH 9.

Figure S5. Replicates and calculations for the substrate independence of the observed AtYoeB-mediated DNase activity.

Figure S6. Original images for data presented in Figure 3.

Figure S7. *In vitro* DNase activity is detected at concentrations as low as 156 nM AtYoeB.

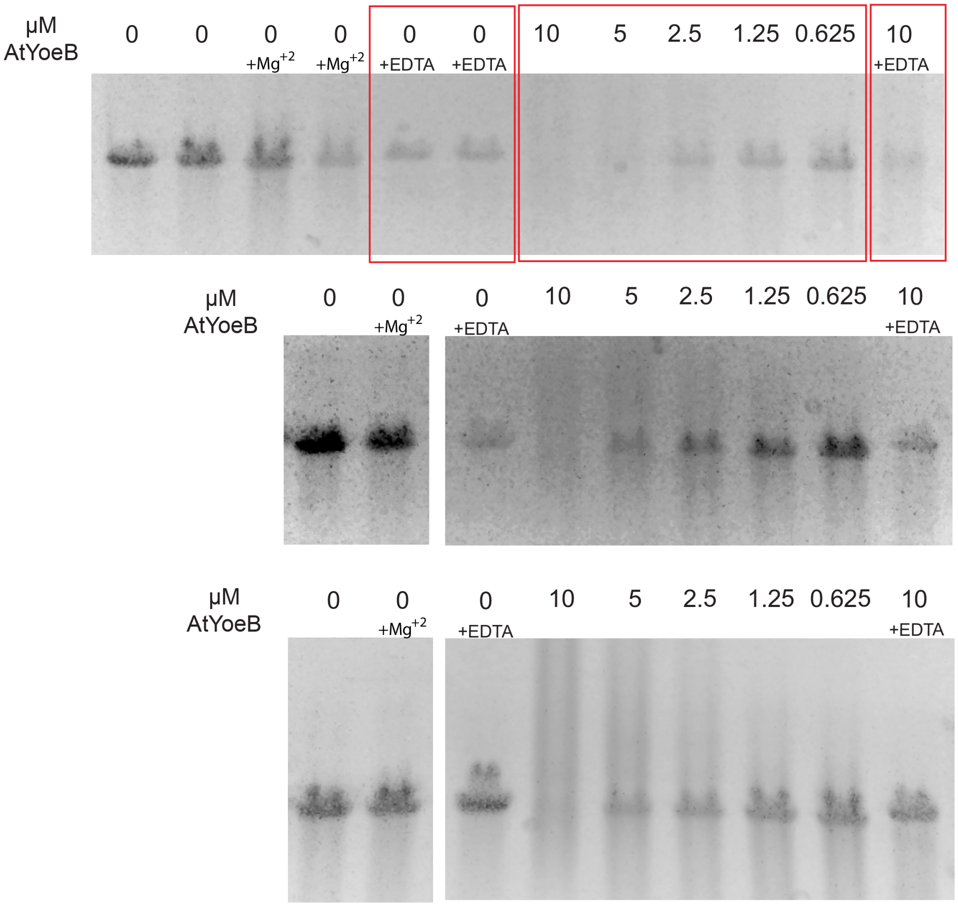

	RNA		
	B:Y1	B:Y2	B:Y3
0 µM	100.0		
0.625 µM	73.1	76.1	50.1
1.25 µM	46.5	45.6	56.5
2.5 µM	25.4	36.1	38.5
5 µM	18.3	14.7	18.1
10 µM	5.1	2.1	12.1

Figure S1. Replicates for the dose dependence of the observed AtYoeB-mediated RNase activity in the absence of the ribosome, also demonstrating a lack of metal dependence. Reactions utilized 80 ng of RNA incubated with increasing concentrations of AtYoeB, and non-specific degradation was visualized by measuring the loss of intensity of the RNA as compared to the control. Magnesium was included as noted at 2.5 mM, while EDTA was present where indicated at 5 mM. *Red boxes indicate images used in Fig. 1a, where the middle panel was flipped horizontally to present lowest to highest concentrations of AtYoeB from left to right.*

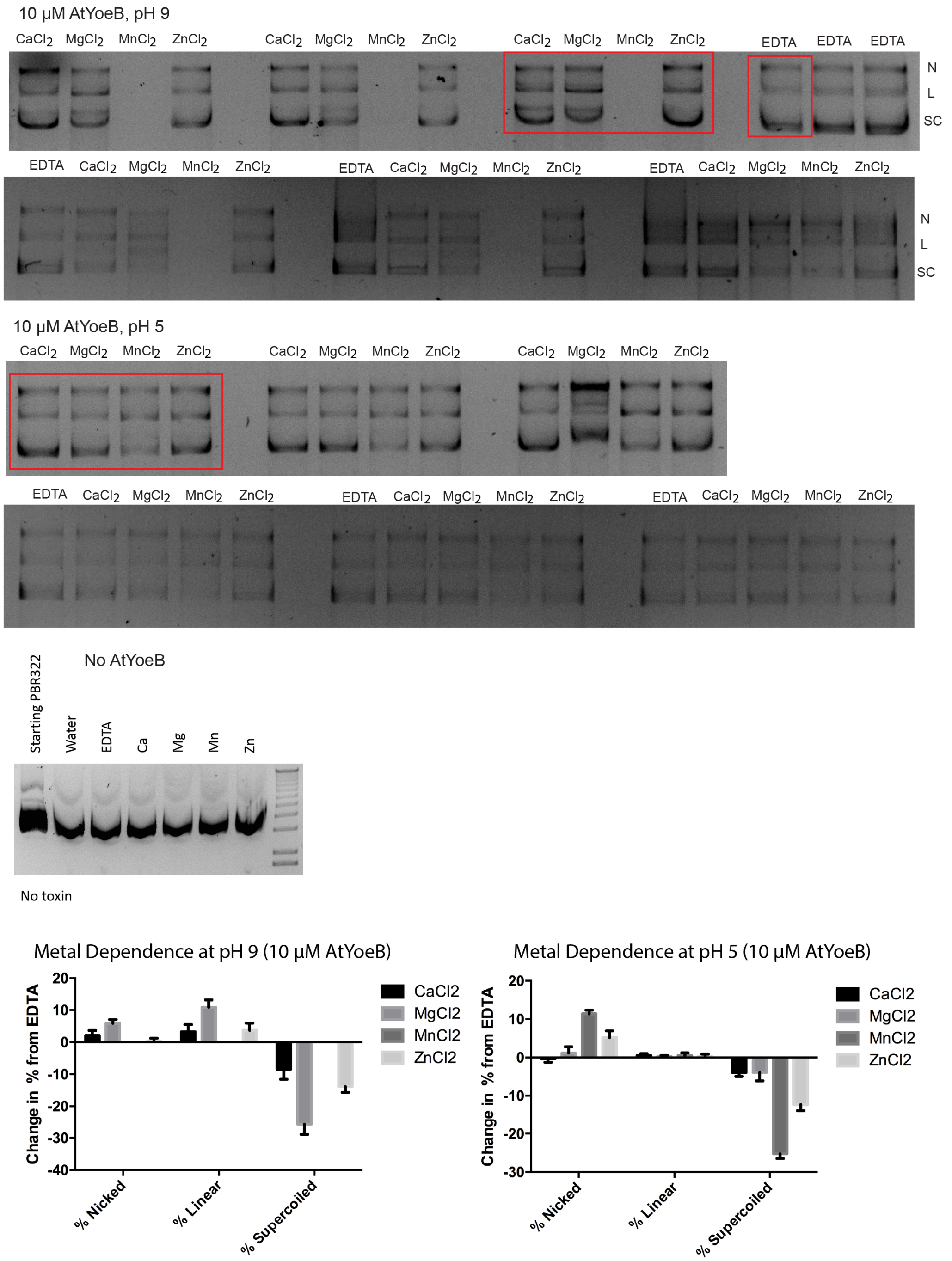

Figure S2. Replicates and calculations for the divalent cation dependence of the observed AtYoeB-mediated DNase activity. $MgCl_2$, $MnCl_2$, $CaCl_2$, or $ZnCl_2$ were added to the DNase assay at a final concentration of 2 mM. *Red boxes indicate images used in Fig. 2a.*

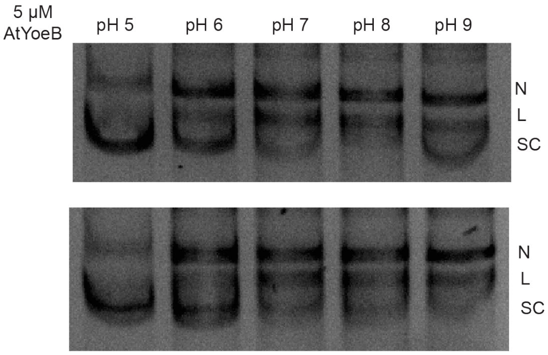

X	Group A			Group B			Group C		
pH	Supercoiled			Nicked			Linear		
X	A:Y1	A:Y2	A:Y3	B:Y1	B:Y2	B:Y3	C:Y1	C:Y2	C:Y3
5	79.1	52.8	60.2	20.9	36.4	34.0	0.0	10.8	5.8
6	52.9	29.5	40.9	40.7	54.5	48.5	6.5	15.9	10.6
7	52.4	11.8	17.4	38.6	51.0	51.3	9.0	37.2	31.3
8	17.6	11.0	15.5	50.4	59.9	54.1	32.1	29.2	30.4
9	9.5	18.2	8.4	60.2	59.2	61.6	30.4	22.6	30.1

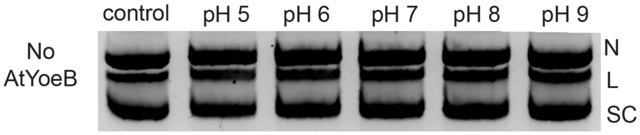

Figure S3. Replicates and calculations for the pH dependence of the observed AtYoeB-mediated DNase activity demonstrate the highest activity is at pH 7 and greater. Bis-Tris buffer at pH 5 or 6 or Tris-HCl at pH 7, 8 or 9 were added to the DNase assay a final concentration of 100 mM. No DNA cleavage is detected in control samples that lack the AtYoeB protein but contain the same buffer components, including Mg^{2+}. *Note: the third replicate is shown in the text Fig. 2b.*

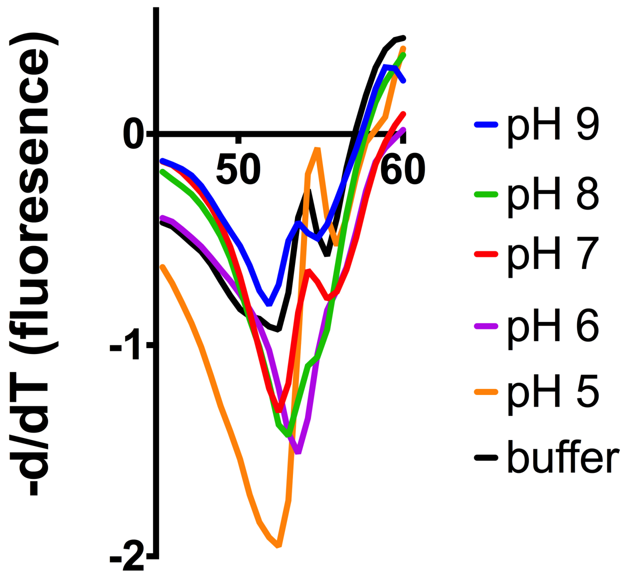

Figure S4. AtYoeB protein is stable at least up to pH 9. The DNA cleavage activity of AtYoeB is maximal at pH 9; the protein appears to remains folded at this pH. This experimental series utilized 10 μM AtYoeB incubated with 100 mM Bis-Tris or Tris at the indicated pH, then subjected to Differential Scanning Flourometry. This technique monitors the fluorescence of SYPRO Orange dye (at a final concentration of "3X"), which quickly increases upon interaction with hydrophobic environments such as the exposed core of an unfolded protein. This increase is converted mathematically to the first derivative, yielding a peak at the transition point. The strength of the signal is not a function of stability, but of subtle changes in overall protein concentration.

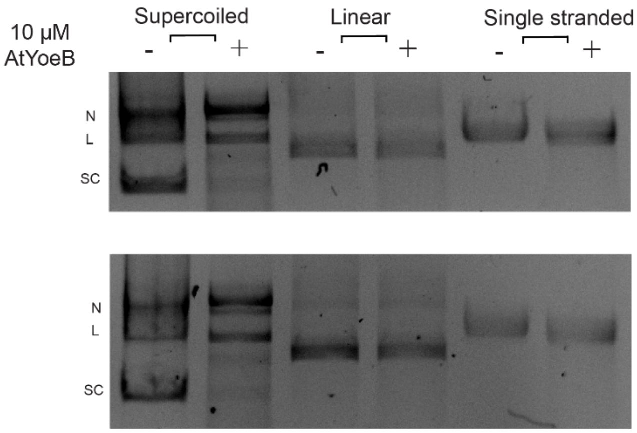

Starting Substrate			Intensity Values			
	**no AtYoeB**			**+ AtYoeB**		
**SC - Nicked band**	8627.271	8536.631	4247.61	9275.045	6923.024	7975.024
**SC - Linear band**	2201.681	406.87	2198.125	5331.933	1300.82	3301.205
**SC - SC band**	14666.401	4515.903	12701.622	866.426	1352.74	990.447
sum	25495.353	13459.404	19147.357	15473.404	9576.584	12266.676
(AtYoeB/sum(noAtYoeB: withAtYoeB))*100				61	71	64
**Linear**	12453.016	10597.054	12453.016	8947.581	7984.711	4744.024
(AtYoeB/sum(noAtYoeB: withAtYoeB))*100				72	75	65
**Single-stranded circular**	12453.016	10597.054	12453.016	8947.581	7984.711	4744.024
(AtYoeB/sum(noAtYoeB: withAtYoeB))*100				74	67	83

Figure S5. Replicates and calculations for the substrate independence of the observed AtYoeB-mediated DNase activity. Substrates were miniprep-purified pBR322, linearized pBR322, or the single-stranded M13 genome (NEB). The extent of degradation for each substrate is approx equal at 30% lost. Further, a minor shift in mobility of ssDNA is mediated by the addition of AtYoeB. *Note: the third replicate is shown in the text Fig. 2c.*

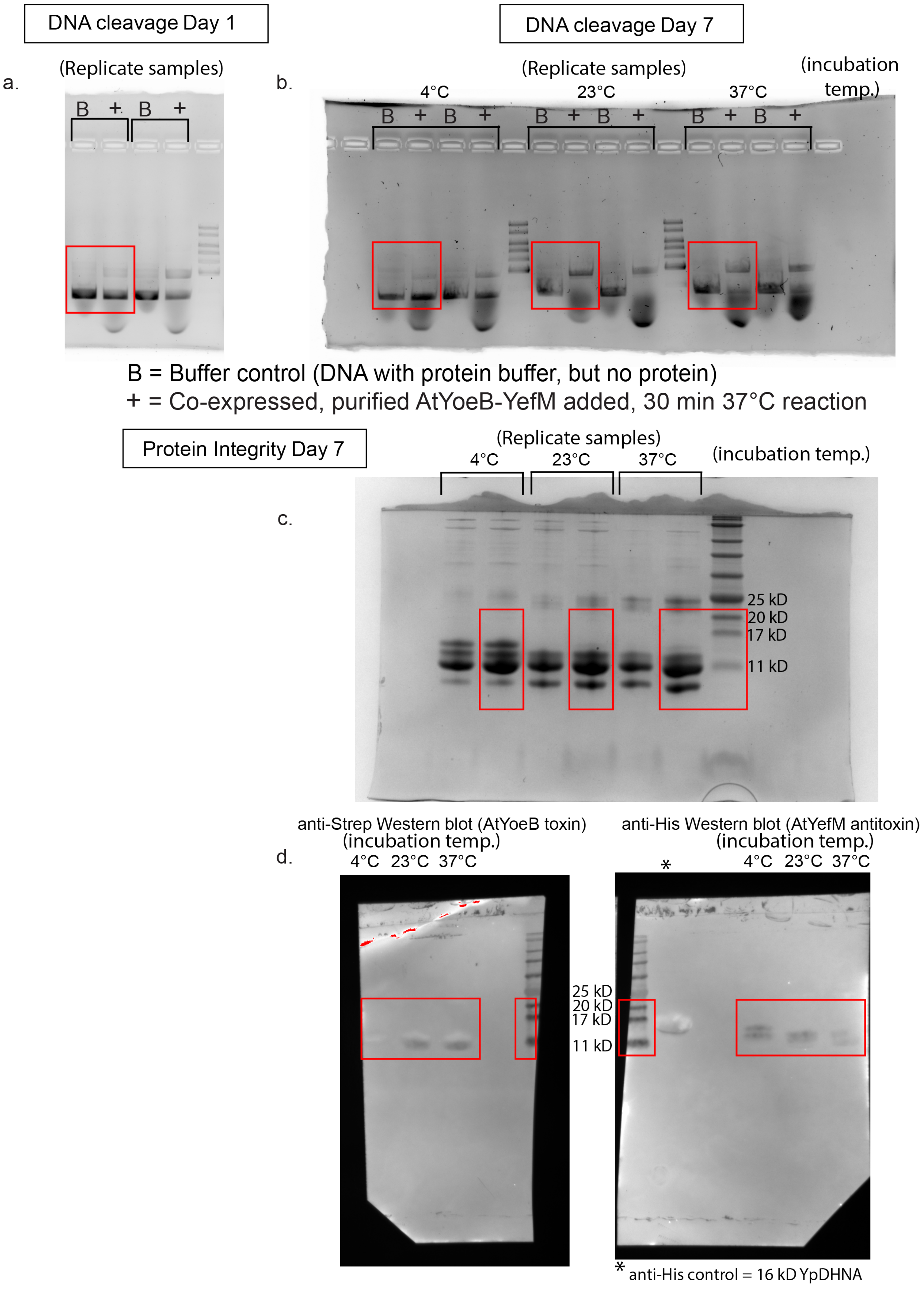
DNA cleavage Day 1
a.
(Replicate samples)
B + B +
DNA cleavage Day 7
b.
(Replicate samples)
(incubation temp.)
4°C
23°C
37°C
B + B + B + B + B + B +
B = Buffer control (DNA with protein buffer, but no protein)
+ = Co-expressed, purified AtYoeB-YefM added, 30 min 37°C reaction
Protein Integrity Day 7
c.
(Replicate samples)
4°C 23°C 37°C
(incubation temp.)
25 kD
20 kD
17 kD
11 kD
d.
anti-Strep Western blot (AtYoeB toxin)
(incubation temp.)
4°C 23°C 37°C
25 kD
20 kD
17 kD
11 kD
anti-His Western blot (AtYefM antitoxin)
(incubation temp.)
*
4°C 23°C 37°C
* anti-His control = 16 kD YpDHNA

Figure S6. Original images for data presented in Figure 3.

DNA cleavage. a. Replicate samples of freshly co-expressed AtYoeB-YefM were incubated with plasmid DNA (pBR322, essentially all supercoiled, as derived from mini-prepped DNA) and incubated for 30 min at 37°C (10 μM protein with 500 ng DNA). Reactions were analyzed on 1% agarose gels in TAE with SYBR safe dye (100 V, 20 min). Control reactions ("B") contained only the "flow-thru" buffer from the centrifugal concentrator used to concentrate the protein complex. **b.** The same reaction performed (in replicates) after incubation of co-expressed AtYeoB-YefM for 7 days at the indicated temperature. *Note*: freshly purified protein was aliquoted into individual tubes and stored at the indicated temperature; DNA samples were added to each ("B" or "+") immediately prior to the 30 min incubation. Lower molecular weight smears in the "+" lanes (with protein) appear due to residual protein binding to the DNA substrate. *Red boxes indicate images used in Fig. 3.*

Protein integrity. c. Replicate aliquots of samples (10 μL at 10 μM) were loaded onto an 18% tris-tricine gel and electrophoresed at 50-100V for approx 100 min. Gels were washed in water for 5 min, then stained with Commassie blue following standard protocols. **d.** The same sample aliquots (10 μL at 10 μM) were loaded on each side of an 18% tris-tricine gel and blotted (as described in the Methods section). The resulting membrane was cut along the lane containing pre-stained markers after blocking but before incubation with primary antibodies. Left side: The toxin maintains a Strep-tag (IBA Lifesciences), allowing specific detection. Right side: The antitoxin contains an N-terminal 6×His tag which is readily detected. A positive control His-tagged protein was included by the ladder for comparison. *Red boxes indicate images used in Fig. 3.*

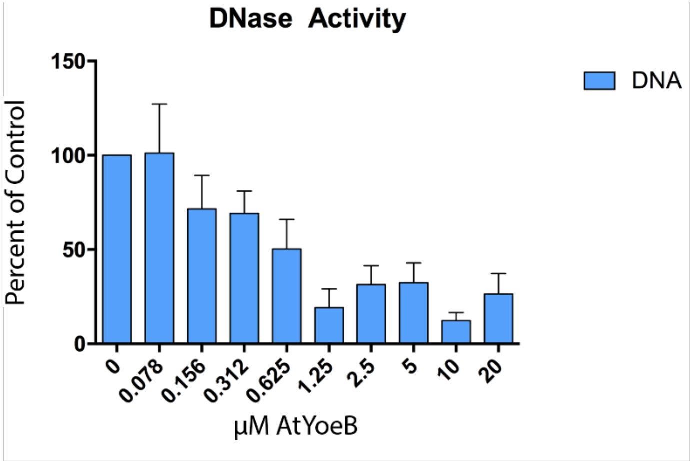

DNA degradation detectable at 156 nM AtYoeB

Figure S7. ***In vitro*** **DNase activity is detected at concentrations as low as 156 nM AtYoeB.**
